# New Biopsy Techniques and Imaging Features of Transrectal Ultrasound for Targeting PI-RADS 4 and 5 Lesions

**DOI:** 10.3390/jcm9020530

**Published:** 2020-02-15

**Authors:** Byung Kwan Park, Sung Yoon Park

**Affiliations:** Department of Radiology, Samsung Medical Center, Sungkyunkwan University School of Medicine, Seoul 06351, Korea; sungyoon.park@samsung.com

**Keywords:** prostate adenocarcinoma, transrectal ultrasound, biopsy, Prostate Imaging Reporting and Data System, magnetic resonance imaging

## Abstract

Purpose: To introduce new biopsy techniques and imaging features of transrectal ultrasound (TRUS) for targeting Prostate Imaging Reporting and Data System (PI-RADS) 4 and 5 lesions Methods: TRUS-guided targeted and/or systematic biopsies were performed in 432 men with PI-RADS 4 and 5 lesions following magnetic resonance imaging examination. A TRUS operator who was familiar with the new techniques and imaging features performed lesion detection. Overall and significant cancer detection rates (CDRs) were compared among the men with PI-RADS 4 and 5 lesions. The CDRs in the peripheral and transition zones were compared. Additionally, we assessed whether targeted or systematic biopsies contributed to cancer detection. The standard reference was a biopsy examination. Results: The overall CDRs in the men with PI-RADS 4 and 5 lesions were 49.5% (139/281) and 74.8% (113/151) (*p* < 0.0001); significant CDRs were 33.1% (93/281) and 58.3% (88/151) (*p* < 0.0001); and CDRs in the peripheral and transition zones were 53.6% (187/349) and 78.3% (65/83) (*p* < 0.0001), respectively. Of the 139 men with clinically significant cancer PI-RADS 4 lesions, 107 (77.0%) were diagnosed by targeted biopsy, 5 (3.6%) by systematic biopsy, and 27 (19.4%) by both. Of the 113 men with clinically significant cancer PI-RADS 5 lesions, 97 (85.8%) were diagnosed by targeted biopsy, 3 (2.7%) by systematic biopsy, and 13 (11.5%) by both. Conclusions: Most PI-RADS 4 and 5 lesions can be targeted with TRUS if the new techniques and imaging features are applied.

## 1. Introduction

Transrectal ultrasound (TRUS)-guided systematic biopsy has been used for detecting prostate cancer in men with high prostate-specific antigen (PSA) levels or a positive digital rectal examination [1,2,3]. However, a substantial number of prostate cancers may remain undetected even with repeated TRUS-guided systematic biopsies [4,5,6]. Moreover, Gleason scores (GSs) obtained from TRUS-guided systematic biopsies frequently underestimate prostate cancers compared with those obtained from prostatectomies [7,8,9]. Hence, magnetic resonance imaging (MRI)–TRUS fusion biopsy [10,11] and in-bore MRI-guided biopsy [12] have been introduced to precisely target significant cancers with fewer cores.

Recently, the Prostate Imaging Reporting and Data System version 2 (PI-RADS v2) has been introduced in clinical practice [13,14]. The PI-RADS helps improve MRI interpretation by stratifying the risk of prostate cancer. PI-RADS 4 and 5 lesions are clearly visible on T2-weighted imaging or diffusion-weighted imaging. These lesions indicate that significant prostate cancer is highly or very highly likely to be present. However, whether a PI-RADS 4 or 5 lesion is visible on TRUS remains to be determined. If these lesions are visible on TRUS, TRUS-guided targeted biopsy can reduce the necessity of MRI–TRUS fusion or in-bore MRI-guided biopsy, because directly targeting a lesion with TRUS is much easier. Nevertheless, the feasibility of TRUS for targeting PI-RADS 4 and 5 lesions has not yet been completely assessed. Accordingly, we hypothesize that PI-RADS 4 and 5 lesions are visible on TRUS and can be targeted without relying on fusion software. The purpose of this study was to introduce new techniques and imaging features of TRUS for targeting PI-RADS 4 and 5 lesions. 

## 2. Methods

### 2.1. Patient Selection

**Figure 1:**
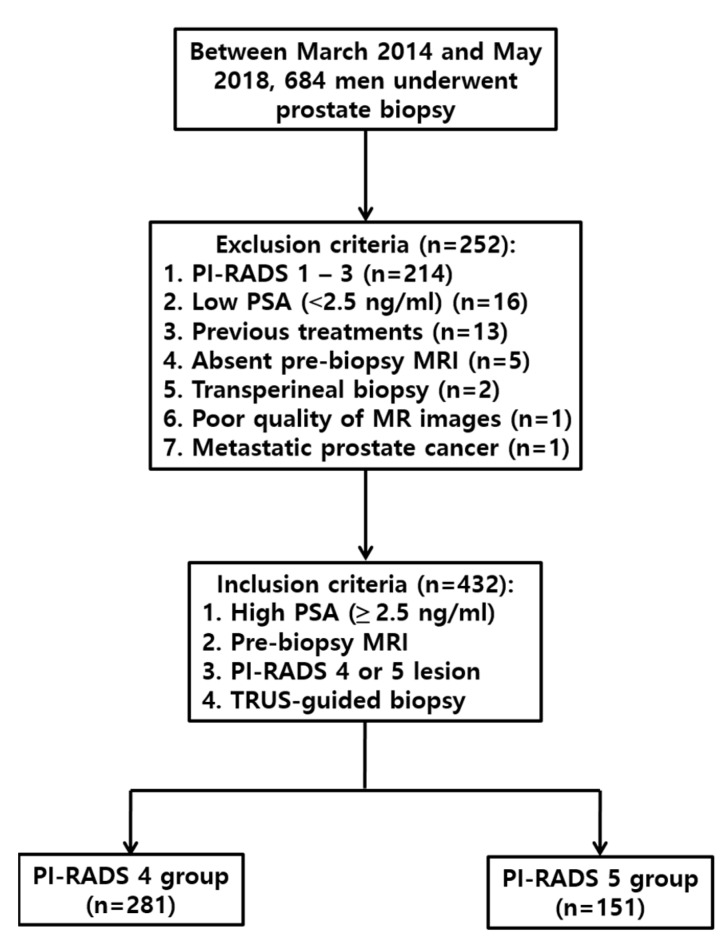
Flow diagram of the study population.

Between March 2014 and May 2018, prostate biopsies were performed in 684 men by a radiologist with 16 years of experience with genitourinary intervention (Figure 1). The inclusion criteria were high PSA level (2.5 ng/mL or higher), PI-RADS 4 or 5 lesions, pre-biopsy MRI, and TRUS-guided biopsy. The exclusion criteria were PI-RADS 1–3 lesions (*n* = 214), low PSA level (*n* = 16), previous treatments (*n* = 13), no pre-biopsy MRI (*n* = 5), transperineal biopsy (*n* = 2), poor-quality MRI images (*n* = 1), and secondary prostate cancer (*n* = 1). A total of 432 men (median age, 67 years; age range, 35–86 years) were included for analysis (Table 1). The median PSA level and PSA range were 5.3 ng/mL and 2.5–2564 ng/mL, respectively. Research involving human participants and/or animals: This study was retrospectively performed on human participants. Informed consent: This retrospective study was approved by our IRB, and the approval number is 2018-10-052-002. The need for informed consent was waived. All data were available for review. 

### 2.2. MRI Interpretation and Biopsy Techniques

Multi-parametric MRI was performed prior to biopsy, with time intervals of 0 to 436 days (median, 39.5 days). A single radiologist evaluated T2-weighted images, diffusion-weighted images, apparent diffusion coefficient map images, and dynamic contrast-enhanced axial MR images prior to the biopsy. Lesion were categorized as 4 (*n* = 281) or 5 (*n* = 151) based on PI-RADS v2 [14]. After interpreting the MR images, the radiologist performed TRUS-guided biopsy. Before he began this study, he had performed TRUS-guided systematic biopsies more than 3000 times and cognitive biopsies more than 500 times according to the pre-biopsy MRI findings [15].

**Figure 2:**
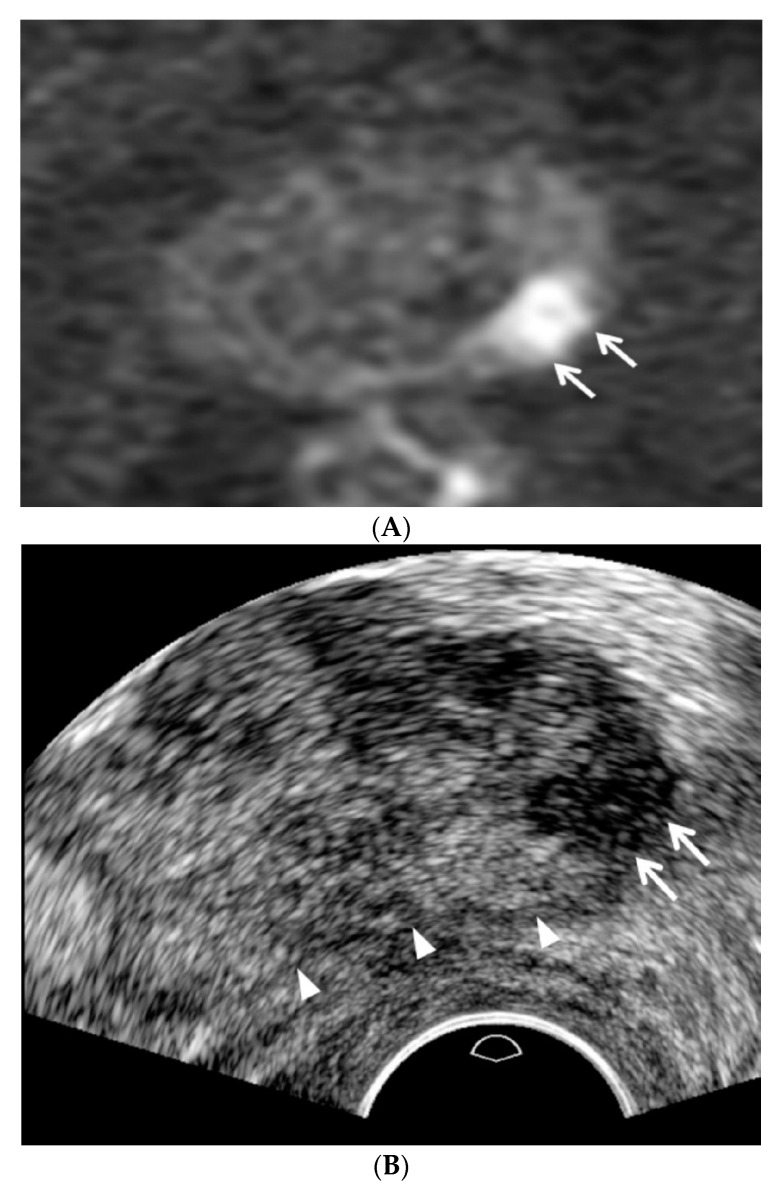
A PI-RADS 4 peripheral cancer in a 70-year-old man. (**A**) Diffusion-weighted axial MR image shows a hyperintense peripheral lesion (white arrows) in the left mid-gland measuring 1.3 cm, suggesting that the PI-RADS lesion category is 4. The patient’s PSA level was 3.56 ng/mL prior to biopsy. (**B**) TRUS axial image clearly shows a hypoechoic peripheral lesion (white arrows) in the left base. The TRUS lesion is located more superiorly to the MRI lesion. Moreover, white arrowheads indicate that the prostate is not compressed by a TRUS probe. TRUS-guided targeted biopsy alone was performed with four cores. All confirmed a Gleason score of 7 (4 + 3) adenocarcinomas.

**Figure 3:**
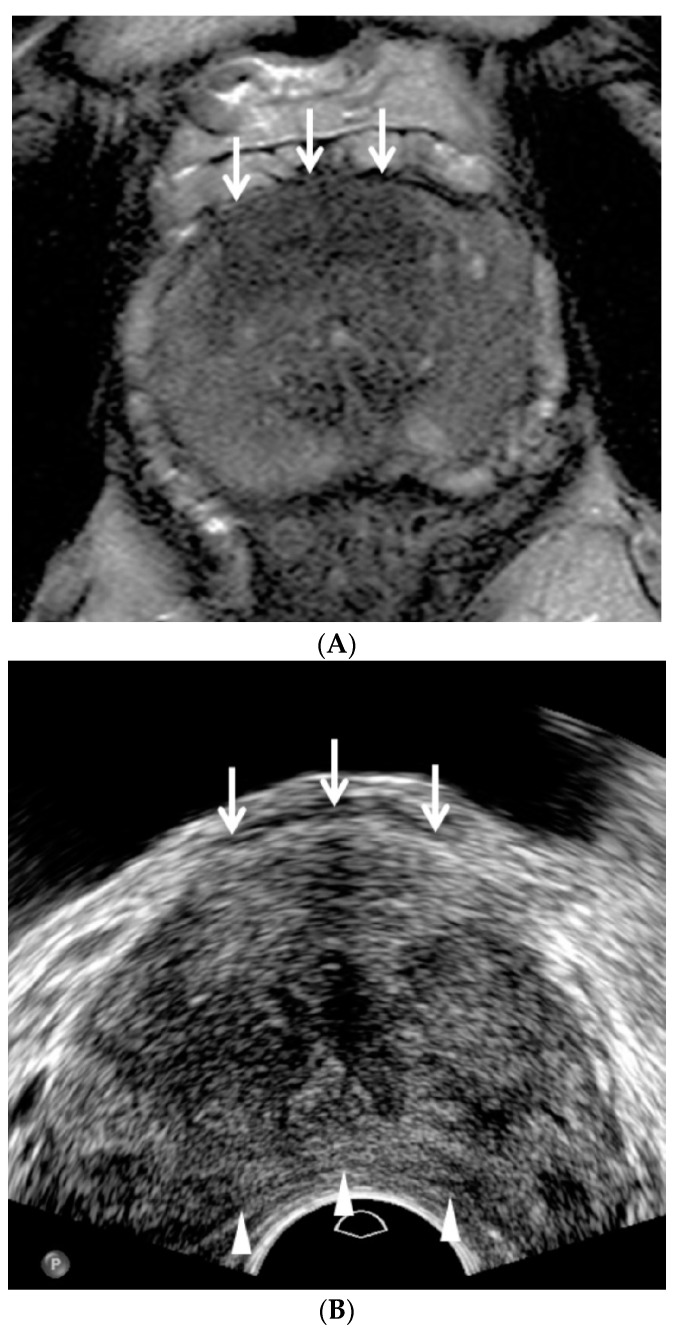
A PI-RADS 5 transition cancer in a 61-year-old man. (**A**) T2-weighted axial MR image shows a moderately hypointense transition lesion (white arrows) in the anterior midline base measuring 2.7 cm, suggesting that the PI-RADS lesion category is 5. The patient’s PSA level was 12.27 ng/mL prior to biopsy. (**B**) TRUS axial image clearly shows a hyperechoic transition lesion (white arrows) in the anterior midline mid-gland. The TRUS lesion is located more inferiorly to the MRI lesion. White arrowheads indicate that the prostate is compressed by a TRUS probe to reduce the tumor-to-probe distance. A TRUS-guided targeted biopsy alone was performed with five cores, in which three and one confirmed Gleason scores of 7 (4 + 3) and 6 (3 + 3) adenocarcinomas, respectively.

All patients had antibiotic medication prior to the biopsy procedure. The patients were placed in the left lateral decubitus and knee chest position, and a high-resolution transducer (C8-4v, Philips Health Care, Bothell, WA, USA) in an ultrasound scanner (IU22, Philips Health Care, Bothell, WA, USA) was introduced transrectally by a single radiologist who interpreted the pre-biopsy MRI according to PI-RADS v2. The transducer was lightly pressed against Denonvillier’s fascia without compressing the prostate to detect focal lesions, which were previously categorized as PI-RADS 4 or 5 on MRI (Figure 2). Prostate compression was performed to reduce the tumor-to-probe distance when an anterior lesion was evaluated and biopsied (Figure 3).

A TRUS operator who was familiarized with the following TRUS techniques and imaging features performed the procedure. First, fundamental US was used instead of harmonic US. Second, the dynamic range of TRUS was maintained less than 50. Third, the prostate was not compressed until PI-RADS 4 or 5 was detected. Fourth, a TRUS lesion was located more superiorly than an MRI lesion as it was closer to the posterior capsule (Figure 2). Fifth, a TRUS lesion was located more inferiorly than an MRI lesion as it was closer to the anterior capsule (Figure 3). Sixth, peripheral and transition cancers appeared to be hypoechoic and hyperechoic, respectively (Figure 2 and Figure 3). Finally, higher-scoring PI-RADS lesions became more hypoechoic or hyperechoic (Figure 2 and Figure 3).

According to the lesion visibility on TRUS, PI-RADS 4 and 5 lesions were re-designated as “clearly visible” and “partially visible.” “Clearly visible” was defined when the margin of a PI-RADS 4 or 5 lesion was completely visible on TRUS. “Partially visible” was defined when the margin of a PI-RADS 4 or 5 lesion was not completely visible on TRUS. If an MRI lesion was clearly visible on TRUS, targeted biopsy alone was performed and the number of cores ranged from 1 to 10 (median, 6; Figure 2 and Figure 3). If an MRI lesion was partially visible on TRUS, systematic biopsy was performed along with targeted biopsy. The number of systematic cores ranged from 3 to 12 (median, 6). If a MRI lesion was invisible on TRUS, a 12-core systematic biopsy alone was performed. When multiple PI-RADS 4 or 5 lesions were detected on MRI, up to three lesions (median, 1; range 1–3) were targeted with TRUS. However, imaging and histological data were analyzed only if index lesions appeared on MRI [14].

### 2.3. Data Analysis

MRI images scanned before 2015 were reassessed using PI-RADS v2. From 2015, when PI-RADS version 2 was released, the original PI-RADS scores categorized by a TRUS operator prior to biopsy were used for analysis.

For demographic comparisons, age, PSA level, prostate volume, PSA density, MRI–biopsy interval, and lesion size were compared between the groups. The CDR was calculated as the number of cancer cases divided by the total number of cases in each group. Significant CDR was defined as the number of significant cancer cases with a GS of 7 or higher divided by the number of all cases in each group.

The overall and significant CDRs in the total population and in each subgroup were obtained for comparison, respectively. The overall and significant CDRs in the peripheral and transition zones were also obtained for the total population and each subgroup for comparison, respectively. Positive core rate was defined as the number of cancer cores divided by the total number of biopsy cores in each group. These CDRs or positive core rates were compared between the PI-RADS groups and among the types of biopsies. The biopsy proportions according to the types were recorded for comparison in each group.

A histological examination was performed to determine which type of biopsy yielded a higher GS or was sampled with a greater number or length of cores with the highest GS in each group undergoing both targeted and systematic biopsies. Furthermore, the number of biopsy or cancer cores, positive core rate, and percentage of cancer core length in cores with the highest GS were also compared. The post-biopsy hospitalization rate and types of complications were also recorded. The total cancer length was divided by the total length of cores obtained.

### 2.4. Statistical Analysis

A Mann–Whitney *U* test was used to compare age, PSA level, prostate volume, PSA density, MRI–biopsy interval, and lesion size between the groups. Fisher’s exact test was used to compare the CDRs, positive core rates, and proportions of biopsies. Wilcoxon’s matched-pairs signed-rank test was used to compare GSs, number of biopsy cores, number of cancer cores, and percentage of cancer core length. Commercial software (PASW Statistics, version 20.0; Chicago, IL, USA) was used for statistical analysis. A two-sided *p* value of <0.05 was considered statistically significant.

## 3. Results

Regarding patient demographics, the PI-RADS 5 group had significantly older age (*p* = 0.0038), higher PSA levels (*p* < 0.0001), greater PSA density (*p* < 0.0001), shorter MRI–biopsy intervals (*p* = 0.0003), and larger lesion sizes (*p* < 0.0001) than the PI-RADS 4 group (Table 1). The prostate volumes appeared to be larger in the PI-RADS 4 group than in the PI-RADS 5 group, but the difference between the groups was not significant (*p* = 0.0748; Table 1).

Among the 432 patients, the overall and significant CDRs were 58.3% (252/432) and 71.8% (181/252), respectively. The overall CDRs of the PI-RADS 4 and 5 groups were 49.5% (139/281) and 74.8% (113/151) (*p* < 0.0001), respectively. The significant CDRs of the PI-RADS 4 and 5 groups were 33.1% (93/281) and 58.3% (88/151), respectively (*p* < 0.0001).

The overall CDRs in the peripheral and transition zones were 53.6% (187/349) and 78.3% (65/83), respectively (*p* < 0.0001). Significant CDRs in these zones were 39.3% (137/349) and 53% (44/83), respectively (*p* = 0.0259). For the PI-RADS 4 group, overall and significant CDRs were 47.3% (124/262) and 78.9% (15/19) in the peripheral zone and 32.1% (84/262) and 47.4% (9/19) in the transition zone, respectively (*p* = 0.0087 and *p* = 0.2057, respectively). For PI-RADS 5 group, overall and significant CDRs were 72.4% (63/87) and 78.1% (50/64) in the peripheral zone and 60.9% (53/87) and 54.7% (35/64) in the transition zone, respectively (*p* = 0.4540 and *p* = 0.5052, respectively).

The proportion of targeted biopsies was significantly higher in the PI-RADS 5 group (*p* < 0.0001), whereas the proportion of targeted and systematic biopsies was significantly higher in the PI-RADS 4 group (*p* < 0.0001; Table 2). Targeted biopsies and targeted and systematic biopsies in the PI-RADS 5 group achieved higher overall CDRs than those in the PI-RADS 4 group (*p* = 0.0001 for both; Table 3). Significant CDRs of targeted and targeted and systematic biopsies in the PI-RADS 5 group were higher than those of targeted and targeted and systematic biopsies in the PI-RADS 4 group (*p* = 0.0009 and *p* = 0.0479, respectively; Table 3).

Of the 281 biopsies in the PI-RADS 4 group, 151 (53.7%) were targeted biopsies, 128 (45.6%) were targeted and systematic biopsies, and 2 (0.7%) were systematic biopsies. The overall CDRs of targeted and targeted and systematic biopsies were 57.0% (86/151) and 41.4% (53/128) (*p* = 0.0116), respectively. The significant CDRs of targeted and targeted and systematic biopsies were 43.0% (65/151) and 21.9% (28/128; *p* = 0.0002), respectively. Of the 139 men with confirmed cancer with PI-RADS 4 lesions, 107 (77.0%) were diagnosed by targeted biopsy, 5 (3.6%) by systematic biopsy, and 27 (19.4%) by both.

Among the 53 men with confirmed cancer who underwent both targeted and systematic biopsies in the PI-RADS 4 group, 21 were diagnosed by targeted biopsy, 5 by systematic biopsy, and 27 by both. Targeted biopsy was superior to systematic biopsy in the overall CDR (*p* = 0.0005), significant CDR (*p* = 0.0008), number of cancer cores (*p* = 0.0003), positive core rate (*p* < 0.0001), and percentage of cancer core length (*p* = 0.0022). The highest GS of targeted biopsies was slightly higher than that of systematic biopsies, but the difference was not significant (*p* = 0.084). The number of biopsy cores with targeted biopsy did not differ from that with systematic biopsy (*p* = 0.7216).

Of the 151 biopsies in the PI-RADS 5 group, 116 (76.8%) were targeted biopsies and 35 (23.2%) were targeted and systematic biopsies. The PI-RADS 5 group had a greater number of targeted biopsies than the PI-RADS 4 group (*p* < 0.0001), whereas the former had a smaller number of targeted and systematic biopsies than the latter (*p* < 0.0001). Systematic biopsy alone was not performed in any case. The overall CDRs of the targeted and targeted and systematic biopsies were 79.3% (92/116) and 60.0% (21/35; *p* = 0.0270), respectively, whereas the significant CDRs of the targeted and targeted and systematic biopsies were 63.8% (74/116) and 40.0% (14/35) (*p* = 0.0183), respectively. Of the 113 men with confirmed cancer with PI-RADS 5 lesions, 97 (85.8%) were diagnosed using targeted biopsy, 3 (2.7%) using systematic biopsy, and 13 (11.5%) using both.

Among the 35 men with confirmed cancer who underwent both targeted and systematic biopsies in the PI-RADS 5 group, 19 were diagnosed using targeted biopsy, three using systematic biopsy, and 13 using both. Targeted biopsy was superior to systematic biopsy in terms of the number of cancer cores (*p* = 0.0034) and the positive core rate (*p* < 0.0001). Targeted biopsies did not differ from systematic biopsies in terms of the overall CDR (*p* = 0.8112), significant CDR (*p* = 0.7972), highest GS (*p* > 0.9999), number of biopsy cores (*p* = 0.7983), or percentage of cancer core length (*p* = 0.1748).

The post-biopsy hospitalization rate was 2.54% (11/432). Eight (1.85%) patients developed acute prostatitis, two (0.46%) developed acute urinary retention, and one (0.23%) developed sepsis.

## 4. Discussion

We hypothesized that high hypo- or hyper-echogenicity indicates more significant cancer. TRUS findings and techniques are important in targeting PI-RADS 4 or 5 lesions. Differences between the scan planes of MRI and TRUS result in differences in the size, shape, and location of PI-RADS 4 and 5 lesions. As a result, there is an obvious image misregistration between MRI and TRUS or image deformation when fusion software is used.

Moreover, the prostate was not compressed until a PI-RADS 4 or 5 lesion was detected. Prostate compression increases the difficultly of detecting a lesion because it results in lesion deformation. Many radiologists and urologists compress the prostate during a TRUS scan. This TRUS procedure distorts not only the prostate gland but also the PI-RADS 4 or 5 lesions and hampers MRI–TRUS imaging or lesion detection in cognitive biopsies. Additionally, if the imaging features of PI-RADS 4 and 5 lesions on TRUS are known, detection of a lesion will be easier in fusion or cognitive biopsies. Even if MRI–TRUS image fusion is not perfect, radiologists or urologists familiar with typical TRUS findings of PI-RADS 4 and 5 lesions will achieve better lesion detection than those not familiar with these findings.

Our study showed that most PI-RADS 4 and 5 lesions were clearly or partially visible on TRUS. Several factors may be responsible for clear lesion visibility. First, fundamental US was used instead of harmonic US to improve imaging resolution although US artifacts increased. Second, using lower dynamic range also improved tissue contrast. Third, higher PI-RADS scores lead to not only a higher likelihood of significant cancer but also greater lesion conspicuity [16,17,18]. Fourth, lesions on TRUS are superiorly or inferiorly located than on MRI according to the tumor location. Axial MR images are typically perpendicular to the urethra, whereas axial TRUS images are oblique to the urethra [19,20]. Fifth, using TRUS in a technically accurate manner, by not compressing the prostrate with a TRUS probe, will help avoid obscuring posterior peripheral cancer by causing it to become embedded in the compressed parenchyma. Sixth, transition cancer is hyperechoic compared with peripheral cancer. Some studies have suggested that iso- or hyperechoic cancers account for up to 40% of all prostate cancers [21,22,23]; however, these studies did not report the locations of these cancers. Our study showed that most of these hyperechoic cancers were located in the transition zone. Accordingly, PI-RADS 4 and 5 transition lesions were clearly visible on TRUS because they were contrasted to a hypoechoic benign prostatic hyperplasia nodule.

When a PI-RADS 4 or 5 lesion is visible on TRUS, MRI–TRUS fusion or in-bore MRI-guided biopsy is unnecessary for lesion targeting. In a primary diagnostic setup, TRUS has several advantages over MRI, such as real-time imaging, short scan time, and low medical cost. MRI–TRUS fusion software or MRI-compatible biopsy devices incur an additional cost for consumers or patients. Many urologists and radiologists are familiar with TRUS-guided procedures and therefore are able to easily target lesions. However, systematic biopsy procedures differ considerably from targeted biopsy procedures in terms of TRUS techniques. These technical recommendations and imaging features of TRUS are very useful in targeting a PI-RADS 4 or 5 lesion with TRUS.

Most radiologists or urologists use an 8–10 MHz transducer, but some investigators adopt a 29 MHz transducer for TRUS imaging [24,25,26]. Ultra-resolution TRUS makes it possible to detect more detailed findings of prostate cancer. Increasing the resolution may lead to increasingly more precise detection or biopsy of PI-RADS 4 and 5 lesions [24,25,26]. If radiologists or urologists become familiar with these techniques and imaging features of TRUS, the number of TRUS-guided targeted biopsies without relying on pre-biopsy MRI will be increased in patients who have a high risk of significant prostate cancer [25,27]. Then, it will reduce the cost for MRI, which is unnecessary in many patients with PI-RADS 4 or 5 lesions.

Generally, TRUS-guided biopsy following MRI has been considered as a cognitive biopsy procedure based on the assumption that prostate cancer is poorly visible on TRUS [19,20,28]. Cognitive biopsy is used to obtain cores in which a tumor is expected to be located [18]. In contrast, targeted biopsy is used to obtain cores within a tumor that is detected on TRUS [16,17,18]. Therefore, a targeted biopsy is needed to accurately detect a PI-RADS 4 or 5 lesion and directly sample the tumor tissue. TRUS-guided targeted biopsy is superior to TRUS-guided cognitive biopsy because the CDR increases and the number of cores decreases by means of precise tumor targeting. The CDR of cognitive biopsy is not as high as that of in-bore MRI-guided biopsy, which directly targets a lesion [12,19,20]. Consequently, a lack of confidence in precise lesion targeting results in systematic biopsy being required [19,20]. Therefore, systematic biopsy in conjunction with cognitive biopsy is an essential procedure to detect prostate cancer.

Recently, PI-RADS has been introduced to improve the detection and characterization of significant cancers with MRI [11,13,14,29]. Biopsy is recommended for PI-RADS 4 and 5 lesions [13,14], which frequently need definitive treatment rather than active surveillance because they are likely or highly likely to indicate significant cancer. Our significant CDRs using TRUS-guided targeted biopsy were not inferior to those reported in the previous studies [11,12,30,31,32] using cognitive, MRI–TRUS fusion, or in-bore MRI-guided biopsy. They showed that significant CDRs were 22.1–78.0% for PI-RADS 4 lesions and 72.4–90.7% for PI-RADS 5 lesions [11,12,30,31,32].

Our study had some limitations. First, the morphologic features of PI-RADS 4 and 5 lesions, other than tumor echogenicity (hypoechoic versus hyperechoic), were not fully evaluated using TRUS. These features include tumor shape (oval versus round), margin (ill-defined versus well-defined), contour (smooth versus irregular), and echotexture (homogeneous versus heterogeneous). Second, our study did not clearly determine whether a systematic biopsy is necessary in addition to a targeted biopsy. The number of men undergoing both biopsies was relatively small in each group. For this reason, target and systematic biopsies could not be compared in PI-RADS 4 or 5 which was clearly seen on TRUS. We thought that good TRUS depiction of this PI-RADS group could skip systematic biopsy. Further investigation is necessary to show how systematic biopsies influence on cancer detection rate in PI-RADS 4 or 5 which is clearly visible on TRUS. Third, our study did not clearly determine the optimal number of cores for cancer detection in targeted biopsy. Fourth, the study design was retrospective. Fifth, we did not differentiate between tumors with different partially visibility.” Therefore, both 10% partially visible tumors and 90% partially-visible tumors were considered as the same group. Sixth, the number of cores and the decision to perform systematic biopsies were not consistent. These factors generally depend on the operator’s experience or technique in performing TRUS procedures. If he or she has a lot of experience or excellent technique when performing TRUS imaging, a tumor will be more easily or frequently detected and the number of biopsy cores or additional systematic biopsies required will be decreased. Seventh, without performing systematic biopsy in patients undergoing targeted biopsy alone, the comparison of CDRs in targeted versus systematic biopsies is difficult. We cannot exclude the likelihood of significant cancer in PI-RADS 1 lesions, which is normal on MRI. From this perspective, in-bore MRI-guided biopsy should be performed for PI-RADS 1 lesions as well as PI-RADS 4 and 5 lesions. This limitation is considered as an intrinsic error. Adding an unnecessary systematic biopsy may increase the complication rate in patients with a PI-RADS 4 or 5 lesion in which significant cancer is sufficiently confirmed with only a few target cores. Finally, biopsy GSs were not compared with prostatectomy GSs because many non-surgical treatments were performed.

## 5. Conclusions

Most PI-RADS 4 or 5 lesions can be targeted with TRUS if the operator gains familiarity with the new TRUS biopsy techniques and imaging features, such as using fundamental US and lower dynamic range, avoiding prostate compression for lesion detection, understanding that the lesion locations are different in MRI and TRUS, and understanding that transition cancers are hyperechoic.

## Figures and Tables

**Table 1:** Patient demographics.

Demographics	PI-RADS Groups		*p* Values
	4 (*n* = 281)	5 (*n* = 151)	
Age (years)	66 (38–83)	68 (35–86)	0.0038
PSA (ng/mL)	4.7 (2.5–21.9)	7.2 (2.5–2564)	<0.0001
volume (mL)	38 (10–193)	34 (13–203)	0.0748
PSAD (ng/mL^2^)	0.12 (0.04–0.68)	0.21 (0.05–65.6)	<0.0001
MRI–biopsy interval (d)	49 (0–436)	29 (0–397)	0.0003
Lesion size (mm)	10 (2–14)	19 (15–57)	<0.0001

MRI, magnetic resonance imaging; PI-RADS, Prostate Imaging Reporting and Data System; PSA, prostate-specific antigen; PSAD, prostate-specific antigen density.

**Table 2:** Proportion of transrectal ultrasound-guided biopsies according to lesion visibility.

Proportion of Biopsy Types	PI-RADS Groups		*p* Values
	4 (*n* = 281)	5 (*n* = 151)	
Targeted biopsy alone (%)	53.7 (151/281)	76.8 (116/151)	<0.0001
Targeted and systematic biopsies (%)	45.6 (128/281)	23.2 (35/151)	<0.0001
Systematic biopsy alone (%)	0.7 (2/281)	0 (0/151)	0.5442

PI-RADS, Prostate Imaging Reporting and Data System.

**Table 3:** Cancer detection rates of the PI-RADS 4 and 5 lesion groups according to the type of biopsy.

Cancer Detection Rates According to the Types of Biopsy	PI-RADS Groups		*p* Values
	4 (*n* = 281)	5 (*n* = 151)	
**Overall cancer detection rates**			
Targeted biopsy (%)	60.0% (86/151)	79.3% (92/116)	0.0001
Targeted and systematic biopsies (%)	41.4% (53/128)	60.0% (21/35)	0.0001
**Significant cancer detection rates**			
Targeted biopsy (%)	43.0% (65/151)	63.8% (74/116)	0.0009
Targeted and systematic biopsies (%)	21.9% (28/128)	40.0% (14/35)	0.0479

PI-RADS, Prostate Imaging Reporting and Data System.
